# Gender differences in preferences for mental health apps in the general population – a choice-based conjoint analysis from Germany

**DOI:** 10.1186/s12888-024-06134-y

**Published:** 2024-10-14

**Authors:** Inga Jagemann, Manuel Stegemann, Ruth von Brachel, Gerrit Hirschfeld

**Affiliations:** 1 School of Business, University of Applied Sciences and Arts Bielefeld, Interaktion 1, 33619 Bielefeld, Germany; 2 Mental Health Research and Treatment Centre Faculty of Psychology, Ruhr University Bochum, Bochum, Germany

**Keywords:** Gender preferences, Discrete choice experiment, Mental health treatment, Artificial intelligence

## Abstract

**Background:**

Men and women differ in the mental health issues they typically face. This study aims to describe gender differences in preferences for mental health treatment options and specifically tries to identify participants who prefer AI-based therapy over traditional face-to-face therapy.

**Method:**

A nationally representative sample of 2,108 participants (53% female) aged 18 to 74 years completed a choice-based conjoint analysis (CBCA). Within the CBCA, participants evaluated twenty choice sets, each describing three treatment variants in terms of provider, content, costs, and waiting time.

**Results:**

Costs (relative importance [RI] = 55%) emerged as the most critical factor when choosing between treatment options, followed by provider (RI = 31%), content (RI = 10%), and waiting time (RI = 4%). Small yet statistically significant differences were observed between women and men. Women placed greater importance on the provider, while men placed greater importance on cost and waiting time. Age and previous experience with psychotherapy and with mental health apps were systematically related to individual preferences but did not alter gender effects. Only a minority (approximately 8%) of participants preferred AI-based treatment to traditional therapy.

**Conclusions:**

Overall, affordable mental health treatments performed by human therapists are consistently favored by both men and women. AI-driven mental health apps should align with user preferences to address psychologist shortages. However, it is uncertain whether they alone can meet the rising demand, highlighting the need for alternative solutions.

## Background

Mental disorders can have significant negative impacts on a range of mental and physical well-being and social and employment outcomes. In 2019, approximately 970 million people around the world were living with a mental disorder, with anxiety and depressive disorders being the most common [1]. Women and men show differences in the factors that contribute to the development of mental disorders and their prevalence. For most internalizing disorders (e.g., major depression and eating disorders), women are more frequently affected, whereas for externalizing disorders (e.g., substance abuse), men are more frequently affected [2, 3]. Gender has been acknowledged by the World Health Organization as a fundamental determinant of mental health and illness, emphasizing the necessity of adopting a gendered mental health practice [4]. Studies demonstrate that socially constructed disparities between genders, encompassing roles, responsibilities, status, and power dynamics, intersect with biological distinctions between the sexes [5, 6]. These interactions have a significant impact on the manifestation of mental health disorders, resulting in distinct preferences for the type and use of mental health treatment options [7–10]. Incorporating patient preferences is a cornerstone of evidence-based practice in psychology [11]. A meta-analysis (52 studies, *n* = 16,000) revealed that the inclusion of patient preferences is not only associated with significantly lower dropout rates but also with significantly better psychotherapy outcomes [12]. The aim of the study was to conduct a choice-based conjoint analysis (CBCA) in order to ascertain gender-based differences in preferences for mental health treatment options, including those utilizing artificial intelligence (AI)-driven mental health applications.

In this study, we assume that the preferences for mental health treatments are influenced by a set of attributes that possess the highest utility. Based on the literature analysis, we selected four attributes (provider, waiting time for feedback, content and costs) for a hypothetical mental health treatment (see Table 1 for the levels of the attributes).

**Table 1:** Sociodemographic characteristics

Characteristics	Total Sample *N* = 2108	Females *N* = 1127 (53.46%)	Males *N* = 981 (46.54%)
**Age**			
18–29 years	384 (18,2%)	233 (20,7%)	151 (15,4%)
30–39 years	431 (20,5%)	225 (20,0%)	206 (21,0%)
40–49 years	440 (20,9%)	219 (19,4%)	221 (22,5%)
50–59 years	423 (20,1%)	229 (20,3%)	194 (19,8%)
60–74 years	430 (20,3%)	221 (19,6%)	209 (21,3%)
**Already used a mental health App**			
Yes	872 (41,4%)	519 (46,2%)>	352 (36,0%)
No	1.219 (57,9%)	605 (53,7%)	614 (62,5%)
I don’t know	17 (0.7%)	3 (0.1%)	14 (1,5%)
**Already used a mental health App**			
Yes	154 (7,3%)	77 (6,8%	77 (7,9%)
No	1.903 (90,3%))	1027 (91,3%)	876 (89,3%)
I don’t know	51 (2,4%)	23 (1,9%)	28 (2,8%)

First, the attribute *provider* was chosen because users currently have the option of choosing between innovative mental health treatments and traditional in-person psychotherapy. This is due to the growing shortage of psychotherapists and psychiatrists worldwide [13–15], leading to an increasing range of digital offerings, such as mental health apps, which can complement or even replace traditional psychotherapy [16, 17]. Furthermore, mental health apps are increasingly incorporating AI to enhance user experience and optimize personalized mental health care [18]. AI is a broad term that refers to various methods and strategies aimed at developing computer systems capable of executing cognitive tasks similar to human capabilities, such as learning, reasoning, problem-solving, pattern recognition, generalization and predictive inference [19]. Previous studies have indicated that numerous users may exhibit reluctance to adopt AI-enabled technologies, particularly within healthcare settings [20–22]. This hesitancy toward innovative treatments is also evident within the mental health domain [23–25]. Previous research on gender differences in innovative mental health treatments has been inconclusive. Lincke et al. [24] found that men have a greater likelihood of engaging in online therapy, while Musiat et al. [25] and Apolinário-Hagen et al. [23] did not find significant associations in their online surveys between gender and the willingness to use internet-based therapies. Gbollie et al. [26] discovered that male participants had a significantly lower likelihood of intending to use mental health apps but a significantly greater likelihood of intending to use mental health chatbots.

Regarding gender differences in traditional in-person psychotherapy, a cross-sectional online study with 115 men and 232 women showed that women liked psychotherapy more than men did and that men liked support groups significantly more than women did [8]. A systematic review (144 studies, *n* = 90.189) revealed that men were more likely to indicate that they found talking to professionals (e.g., psychologists) difficult [9]. In the CBCA, users were presented with the option of choosing between traditional, in-person therapy and AI-driven mental health apps. Given the inconsistent results of previous studies, it would be interesting to examine gender differences in preferences when choosing between AI-driven therapy and traditional psychotherapy.

Second, we included *waiting time for feedback* as an attribute. This addresses the prevalent issue of prolonged waiting periods for appointments in the mental health sector, frequently stemming from a shortage of psychotherapists [13]. In Germany, the waiting period from the initial consultation to the start of therapy is reported to be 142.4 days [27]. A significant advantage of AI-driven mental health apps is that they offer immediate access to treatments, feedback and assistance, thereby overcoming geographical barriers and reducing waiting times for individuals seeking support [28, 29]. Therefore, understanding the decision-making process women and men undergo when faced with the choice between lengthy waiting times for traditional psychotherapists and shorter waiting times for AI-driven mental health apps presents an intriguing area of inquiry.

Third, incorporating *content* tailored to gender-specific preferences in mental health treatment represents a crucial aspect of addressing individualized needs and enhancing treatment outcomes [7, 30, 31]. Previous research has shown that women tend to gravitate toward coping strategies emphasizing social support and emotional expression [7], while men may prefer problem-focused coping methods [8]. Regarding e-mental health programs, women showed a preference for interactive platforms that incorporate practical exercises for stress reduction, along with the provision of high-quality information on work-related stress and guidance from mental health professionals [32]. In contrast, men displayed a preference for certain program aspects, such as coping strategies for stress and work-related issues, to be delivered in a video game format [32]. By considering how gender-specific preferences impact the design of mental health interventions, practitioners can better cater to diverse populations and optimize treatment outcomes. Therefore, a CBCA was conducted to investigate gender differences in content preferences.

Fourth, we integrated the *costs* of mental health treatment as a significant attribute into our analysis. In Germany, mental health apps can undergo a certification process, known as a DiGA certificate, which enables them to be prescribed by physicians or psychotherapists [33, 34]. This certification, introduced in 2019, allows for cost coverage by statutory health insurance, meaning that both traditional psychotherapy and DiGA apps are reimbursed. However, individuals with statutory health insurance also have the option to access private services, which often offer more immediate availability. Nonetheless, individuals opting for private services are required to bear the costs themselves [35]. Thus, the inclusion of costs as an attribute serves to elucidate the willingness of women and men to invest financially in mental health treatment options. This aspect is vital for understanding the broader implications of treatment accessibility and affordability within the German healthcare context.

Due to a lack of consensus on gender differences in mental health treatment options, which have largely been based on questionnaire-based research findings [8, 23–25, 32], this study employs a CBCA to systematically identify these preferences in the general population. CBCA surpasses self-reported questions by revealing implicit preferences and decision-making trade-offs, simulating realistic scenarios to offer insights into actual behavior [36]. To the best of our knowledge, there is currently only one study examining gender-based preferences for mental health treatment options using a CBCA. The discrete choice experiment (*n* = 1984) revealed a strong preference for personal contact with a psychotherapist in blended care, proven effectiveness and low cost [37]. However, the study was not specifically planned to test for gender differences, so it is unknown whether this is due to publication bias or no effect. Our research sought to address the following questions:

RQ1: Is gender related to participants’ individual importances for mental health treatment?

RQ2: Is gender related to participants’ part-worth utilities for mental health treatment?

Moreover, our investigation examined whether age, prior experience with psychotherapy and previous use of mental health apps are associated with participants’ individual importances. Research suggests that older individuals may gravitate toward face-to-face psychotherapists due to a perceived necessity for personal interaction and trust-building [38, 39], while younger individuals may prefer internet-based mental health treatment, valuing convenience and accessibility [24, 32]. Individuals with previous experience in traditional face-to-face psychotherapy may prioritize aspects such as therapist-patient relationships and personalized interactions, increasing the importance placed on face-to-face psychotherapists [37]. Conversely, those with prior experience in utilizing mental health apps may prioritize factors such as anonymity, flexibility, and self-directed therapy, thus favoring AI-driven mental health apps [40, 41]. Thus, we presented the following inquiry:

RQ3: Are age, treatment history and previous mental health app usage related to participants’ individual importances?

Previous studies have focused primarily on attitudes toward online-based therapies in a broad sense, without specifically considering AI as an independent therapeutic modality [23–25, 32, 37]. Consequently, there is an evident imperative to investigate gender differences regarding mental health treatment options that incorporate AI as a treatment option:

RQ4: What factors are related to preferring AI-based treatment to conventional human therapy?

## Method

This cross-sectional study used a CBCA to examine gender preferences for mental health treatment options from the users’ perspective. CBCA is a quantitative marketing research method that is based on the premise that any product or service can be described by its characteristics (attributes) and that the extent to which an individual values a product or service depends on the levels of these characteristics [42]. Participants are asked to choose between different sets of choices, where each set consists of two or more hypothetical products and their predefined attributes, each with a combination of levels. Given a sufficient number of choices per participant, it is then possible to statistically estimate the importance of each attribute and level for the choice in terms of part-worth utilities. The method offers a behavioral approach and is less susceptible to social desirability and other biases [36, 43]. CBCA is increasingly applied to healthcare settings [44, 45] and has great potential for identifying patient preferences to contribute to patient-centered care [46]. Our study is guided by the 10-item checklist for conjoint analysis applications in health care established by the Good Research Practices for Conjoint Analysis Task Force of the International Society for Pharmacoeconomics and Outcomes Research (ISPOR) [47].

### Survey design

**Fig. 1:**
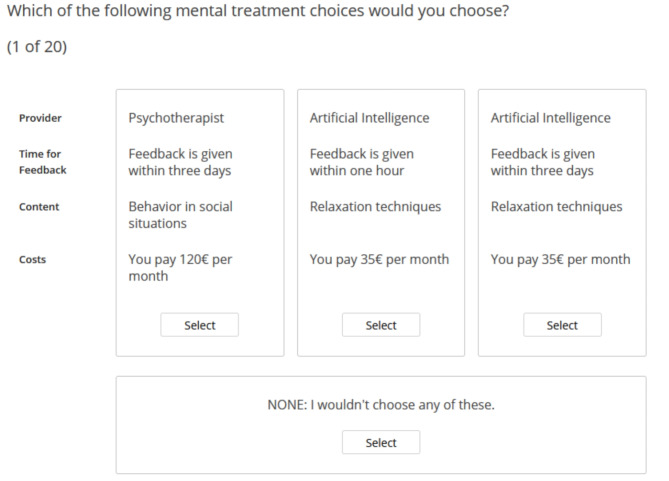
Example choice set (translated from German)

The survey was developed with Sawtooth Software Lighthouse Studio – Version 9.15.0. Informed consent was obtained from all participants before the start of the questionnaire, confirming their agreement to participate in the anonymous study. Then, participants answered sociodemographic questions (age, gender) followed by questions about whether they had already experienced traditional psychotherapy and whether they had already used a mental health app. The gender of the participants was self-reported (female, male). After that, the CBCA tasks were provided. Participants were presented with 20 different choice sets, each consisting of three different modes of mental treatment, that were randomly selected from the potential 36 (2 × 2 × 3 × 3) possible combinations of levels (see Table 1 for the levels). The default Sawtooth algorithm-*balanced overlap method* generated the choice sets. This method occupies an intermediate position between the random selection and complete enumeration strategies. It allows for approximately half as much overlap as the random method [42]. For each set of choices, participants were asked to indicate which mental health treatment they preferred the most. We also included an option for participants to indicate that they would not choose any of the treatments. Figure 1 shows an example of the choice task used in this study. Finally, the survey asked again whether the data could be used for analysis in anonymized form if participants changed their minds during the course of the survey and to filter out people who just wanted to “click through” without seriously answering the questions. To prevent comprehension issues and technical difficulties, the study was pre-tested with twelve university students who volunteered to participate in the trial.

### Sample

Recruitment was based on a nationally representative sample of 2,108 adults from a market research panel. The data were collected from August 24, 2023, through September 10, 2023. To take part in the survey, participants were given an incentive (€1.00). The Psychology Faculty of Ruhr University Bochum has granted ethical approval (874) for this study, and the research was preregistered on aspredicted.org [https://aspredicted.org/6FV_LZV].

A total of 2,827 participants completed the survey. However, 602 participants (21.29%) were excluded from the subsequent analysis due to either completing the survey too quickly (less than three minutes, 578 participants) or too slowly (more than 200 min, 24 participants). The time-based exclusion criteria were informed by a research paper from Sawtooth [48], which indicated that the average completion time for 20 tasks is approximately five minutes. The benchmark was employed to evaluate the response times. A completion time of less than three minutes indicated that the participants may not have adequately processed the questions, whereas a time exceeding 200 min suggested the potential for difficulties or disruptions during the survey. Additionally, 117 participants (4.14%) were excluded due to non-binary identity (22 participants) or failure to provide consent (95 participants). After these exclusions, data from 2,108 participants (74.57%) were analyzed. On average, participants took 8.3 min to complete the survey, with the completion times ranging up to 177.52 min.

### Data analysis

The data were provided by the administrative module (URL) of the Sawtooth Software Lighthouse Studio. Part-worth utilities were estimated using Sawtooth HB (version 9.15.0) to perform a hierarchical Bayes method estimation, running 10,000 iterations and 10,000 draws per participant. Sawtooth uses the *Monte Carlo Markov Chain* algorithm to estimate the Hierarchical Bayes (HB) Mixed Logit (MNL) model. A detailed description of the hierarchical Bayes method and its implementation can be found in the references [49]. The data analysis was carried out in R Studio (version 4.3.1). To address RQ 1 and RQ2, a series of t-tests were conducted to evaluate potential gender differences in the relative importance assigned to the attributes and in the part-worth utilities assigned to the levels. Furthermore, we used ANCOVAs to test for gender differences in the relative importances and in the part-worths utilities (dependent variables). RQ3 was accomplished by fitting linear regression models to examine the relationships between the dependent variables (importances) and the independent variables (age, previous experience with psychotherapy, and previous use of mental health apps). RQ4 was addressed through a two-step process. First, participants were categorized based on their individual part-worths to determine their preference for an AI-based provider. Logistic regression analysis was subsequently used to predict preferences for either human or AI-based providers, controlling for variables such as gender, age, previous experience with psychotherapy and previous use of mental health apps. The data used in this study can be accessed at zenodo.org.

## Results

### Sociodemographic characteristics

Table 2 provides an overview of the socio-demographic characteristics. The sample consisted of 1,112 females (53%) and 981 males (46%). The weighted mean age of the participants was estimated by the weighted arithmetic mean method [50] and was 45.02 years (range 18–74 years). When asked about their previous experience with psychotherapy, 872 participants (41%) had already received mental health treatment, while the majority, 1219 (57%), had not, and 17 (0.8%) were unsure. Participants were queried about their prior use of mental health treatment apps, revealing that 154 participants (7.3%) had previously used such apps, 1903 (90%) had not, and 51 (2%) were unsure. When considering gender differences, it becomes evident that more women (46%) had previously received psychotherapeutic treatment than men (35%). Regarding the previous use of mental health apps, it is noteworthy that there was a balanced distribution between females (6%) and males (7%). However, the majority of participants of both genders had not used a mental health app prior to this study, with 1027 females (91%) and 876 males (89%).

**Table 2:** Mean values (Standard Deviations) of gender differences in Importances

	Female *N* = 1127	Male *N* = 981	*p* ^a^	Effect size	*p* ^a^
Provider	31.9 (17.8)	29.3 (18.1)	0.001	0.144	0.006
Time for Feedback	3.95 (4.88)	4.72 (5.80)	0.001	-0.144	< 0.001
Content	10.7 (10.7)	10.2 (10.4)	0.211	0.055	0.366
Costs	53.4 (18.9)	55.8 (19.5)	0.005	-0.124	0.034

Note: ^*a*^*p* from t-test; ^*b*^*p* from ancova

### Main results

To address RQ1 regarding the importance of provider, waiting time for feedback, content, and costs in participants’ preference for mental health treatment, Table 3 presents a summary of the relative importances (mean values) of these attributes, separated by gender. Overall, costs were the most important attribute for both genders. Costs are rated significantly higher by males than females (55.8 vs. 53.4; *p* < 0.01; *Cohen’s D* = -0.124). Provider ranks as the second important attribute for both genders, with females considering it significantly more important than males (31.9 vs. 29.3; *p* < 0.001; *Cohen’s D* = 0.144). Content is the third most important attribute for both genders, being relatively similar among women (10.7) and men (10.2) (*p* > 0.05; Cohen’s *D* = 0.055). For both females and males, waiting time for feedback was the least important attribute (3.95 vs. 4.72; *p* < 0.001; *Cohen’s D* = -0.144). Men place significantly greater value on waiting time for feedback than women do. Importantly, these gender differences remained stable even after controlling for age and treatment history.

**Table 3:** Part-worth utilities

	female *N* = 1127	male *N* = 981	*p* ^a^	Effect Size	*p* ^b^
Psychotherapist	60.8	54.0	< 0.001	0.164	< 0.001
Artificial Intelligence	-60.8	-54.0	< 0.001	-0.164	< 0.001
Feedback is given within one hour	6.67	8.06	0.007	-0.119	0.006
Feedback is given within three days	-6.67	-8.06	0.007	0.119	0.006
Emotions in social situations	0.19	-1.74	0.017	0.104	0.016
Behavior in social situations	3.38	2.37	0.267	0.048	0.260
Relaxation techniques	-3.57	-0.63	0.061	0.082	0.057
Costs are completely covered by the health insurance	99.6	104	0.059	-0.083	0.053
You pay 120€ per month	-107	-109	0.399	0.037	0.391
You pay 35€ per month	7.62	4.92	0.010	0.113	0.009

Note: ^*a*^*p* from t-test; ^*b*^*p* from ancova

### Exploratory results

To examine RQ2 regarding the relationship between gender and participants’ part-worth utilities for mental health treatment, Table 1 presents a summary of the average zero-centered part-worth utilities for the levels categorized by gender. For both genders, the most preferred form of mental health treatment is performed by a psychotherapist, who focuses on behavior in social situations, offers feedback within an hour, and is fully covered by health insurance. Compared to psychotherapists, AI is less preferred, with men being less averse to AI than women are (-54.0 vs. -60.8; *p* < 0.001; *Cohen’s D* = -0.164). Regarding waiting time for feedback, men prioritize fast feedback more than women do (8.06 vs. 6.67; *p* < 0.01; *Cohen’s D* = -0.119). In regard to content, women tend to prefer behavior in social situations (3.38 vs. 2.37; *p* > 0.05, Cohen’s *D* = 0.0482) and emotions in social situations (0.187 vs. -1.74; *p* < 0.01; *Cohen’s D* = 0.104) more than men do. Relaxation techniques are more likely to be rejected by both genders, with men being less averse than women are (-0.629 vs. -3.57; *p* > 0.05; *Cohen’s D* = 0.0818). Regarding the cost of mental health treatment, both men and women prefer that the cost be fully covered by health insurance, but men are more likely to prefer this (104 vs. 99.6; *p* < 0.05; *Cohen’s D* = -0.0828). In addition, men reject higher costs more than women do.

To explore RQ3 regarding the associations between participants’ individual importances and the independent factors gender, age, previous experience with psychotherapy and prior usage of mental health apps, Table 4 provides an overview of the regression analysis. Regarding gender, a statistically significant association was observed, with males indicating a lower preference for provider (-2.17, *p* < 0.01) and higher importance ratings for costs (1.80, *p* < 0.05) and waiting time (0.82, *p* < 0.001). No significant differences were noted for content (-0.44, *p* > 0.05). Age demonstrated significant associations, indicating that as age increased, there was a decrease in the perceived importance of waiting time (-0.56, *p* < 0.001) and content (-0.77, *p* < 0.001) and an increase in the perceived importance of costs (1.84, *p* < 0.001). No statistically significant differences were observed for age and provider (-0.51, *p* > 0.05). Previous experience with psychotherapy showed significant associations, revealing that participants who had not already received mental health treatment had lower importance ratings for provider (-3.02, *p* < 0.001) and higher ratings for costs (3.24, *p* < 0.001), while showing no difference in ratings for waiting time (0.14, *p* > 0.05) and content (-0.36, *p* > 0.05). The prior use of mental health apps also played a significant role, with users who had not already used mental health apps showing greater importance for provider (4.72, *p* < 0.001) and less importance for waiting time (-2.42, *p* < 0.001) and content (-2.88, *p* < 0.001). No statistically significant differences were found for app usage and costs (0.58, *p* > 0.05).

**Table 4:** Results of the regression analysis predicting relative importances with gender, age, treatment experience, app experience

	Provider			Waiting time			Content			Costs		
*Predictor*	*Est.*	*CI*	*p*	*Est.*	*CI*	*p*	*Est.*	*CI*	*p*	*Est.*	*CI*	*p*
Gender	-2.17	-3.71 – 0.64	0.006	0.82	0.37–1.27	< 0.001	-0.44	-1.35–0.46	0.333	1.80	0.16–3.43	0.031
Age	-0.51	-1.05–0.04	0.070	-0.56	-0.72 – -0.40	< 0.001	-0.77	-1.09 – -0.45	< 0.001	1.84	1.26 – 2.43	< 0.001
Already received a traditional psychotherapy	-3.02	-4.54 – -1.50	< 0.001	0.14	-0.31–0.58	0.547	-0.36	-1.25 – 0.53	0.434	3.24	1.63 – 4.86	< 0.001
Already used a mental health App	4.72	2.22–7.21	< 0.001	-2.42	-3.15 – -1.69	< 0.001	-2.88	-4.34 – -1.42	< 0.001	0.58	-2.08 – 3.24	0.666

Note. Est. = Estimation

In RQ4, we compared participants who preferred AI-based therapy to those who preferred traditional human therapy (Table 5). Overall, only 171 participants (8.11%) preferred AI-based therapy over human therapy. Male participants (*Odds Ratio [OR]* = 1.52; 95% *CI* = 1.11–2.09; *p* < 0.01) and those with previous experience with mental health apps (*OR* = 2.74; 95% *CI* = 1.72–4.24; *p* < 0.001) were more likely to prefer AI over a human, while those with previous experience with psychotherapy were less likely to prefer AI over a human (*OR* = 0.69; 95% *CI* = 0.49–0.96; *p* < 0.05). Age did not affect the preference for AI-based therapy.

**Table 5:** Relationships between gender, Age, Treatment and App and Importances

		*Preference*			
		human therapist	AI	OR (univariable)	OR (multivariable)
Gender	female	1052 (93.3)	75 (6.7)	-	-
	male	885 (90.2)	96 (9.8)	1.52 (1.11–2.09, *p* = 0.009)	1.42 (1.03–1.96, *p* = 0.031)
Age	18–29	359 (93.5)	25 (6.5)	-	-
	30–39	392 (91.0)	39 (9.0)	1.43 (0.85–2.43, *p* = 0.181)	-
	40–49	398 (90.5)	42 (9.5)	1.52 (0.91–2.57, *p* = 0.114)	-
	50–59	386 (91.3)	37 (8.7)	1.38 (0.82–2.36, *p* = 0.235)	-
	60–74	402 (93.5)	28 (6.5)	1.00 (0.57–1.76, *p* = 0.999)	-
Already received a traditional psychotherapy	No	1107 (90.8)	112 (9.2)	-	-
	Yes	815 (93.5)	57 (6.5)	0.69 (0.49–0.96, *p* = 0.029)	0.59 (0.42–0.84, *p* = 0.004)
	I do not know	15 (88.2)	2 (11.8)	1.32 (0.21–4.75, *p* = 0.716)	1.01 (0.15–3.72, *p* = 0.994)
Already used a mental health App	No	1766 (92.8)	137 (7.2)	-	-
	Yes	127 (82.5)	27 (17.5)	2.74 (1.72–4.24, *p* < 0.001)	3.25 (2.00-5.14, *p* < 0.001)
	I do not know	44 (86.3)	7 (13.7)	2.05 (0.83–4.36, *p* = 0.085)	2.24 (0.90–4.82, *p* = 0.056)

## Discussion

The aim of the present study was to elucidate gender differences in preferences for mental health treatment, including AI-driven mental health apps. Highlighting the nuanced differences in gender-based preferences could inform the design and implementation of these technologies. Using a CBCA, we identified strong and consistent preferences for certain treatment options. Costs emerged as the overall strongest driver of participants’ preferences. The linear regression results revealed significant relationships between demographic characteristics. Specifically, males prioritized costs and waiting time more than females but indicated a lower preference for provider, while age, previous experience with psychotherapy and use of mental health apps also played significant roles in shaping preferences. Only a minority of participants preferred AI-based treatment over human therapy. In the following, we discuss these results in turn before discussing some of the limitations of the present work.

With regard to overall importances we found that costs were the most important attribute for both genders, with men placing a greater emphasis on price than women. Both men and women generally prefer mental health treatment to be fully covered by their health insurance. However, men tend to have a stronger preference for lower costs. These differences may be rooted in traditional gender roles and socialization, where men are often conditioned to prioritize efficiency and cost-effectiveness, potentially at the expense of interpersonal factors. Our results support the research conducted by Phillips et al. [37], in which participants highly valued low prices. The characteristics of the German healthcare system may explain the preference for not self-paying, as psychotherapeutic treatments are fully covered for those with statutory health insurance if patients have a clinical indication and authorization [51]. This implies that any introduction of AI-driven mental health apps within this context would need to align closely with the financial expectations and realities of the users to gain acceptance.

Provider was the second most important attribute, with women giving greater priority to provider than men. Both genders strongly prefer traditional face-to-face psychotherapy to receive psychological treatment. Our findings confirm previous research in which participants still preferred traditional mental health treatment over apps or internet-based therapies [23–25, 37]. Women, in particular, have a stronger preference for psychotherapists and are more hesitant toward AI. This gender disparity suggests that women may place greater value on the therapeutic relationship and human connection in treatment, which could be a significant barrier to the adoption of AI-driven therapies among female patients. This finding is consistent with previous research and reinforces the established trend that women are more likely than men to seek psychotherapeutic help when it is offered in person [8]. Furthermore, the reluctance of both genders toward AI as a provider of mental health treatment is in line with the phenomenon of algorithm aversion, where individuals prefer human practitioners, even when AI demonstrates superior performance [20, 52]. In Germany, physicians are preferred over apps, as demonstrated by the use of prescribed apps (DiGA). Currently, most DiGAs (25 out of 55) focus on mental health treatments, but the total number of prescriptions was relatively low from September 2020 to September 2022 [34, 53]. Our sample supports these findings, as more than 90% of participants reported never having used a mental health treatment app. The German Society for Psychotherapy also advises caution in the use of DiGA in psychotherapeutic care and emphasizes the need for a clear distinction between regulated psychotherapy and unregulated mental health apps [54]. This reluctance towards AI-driven apps (and prescriped apps in general) could be mitigated through increased familiarity in less critical areas of healthcare, gradually building trust in AI’s capabilities.

Content was the third most important attribute for both men and women, with their preferences being relatively similar. Both genders are more likely to reject relaxation techniques and prefer behavior or emotions in social situations. However, men exhibit the strongest aversion to mental health treatment that involves dealing with emotions in social situations. These results are in line with previous findings that women are more likely than men to express their emotions [7]. Additionally, our findings corroborate previous research on mental health app feature preferences, indicating that women are more likely to focus on their emotions by favoring apps that offer activities and techniques aimed at alleviating symptoms of stress and depression [32]. Such findings are important for the design and marketing of mental health apps.

Waiting time for feedback is the least important attribute for both genders, with men placing a slightly greater value on waiting time than women. Despite the touted benefits of instant accessibility, support, and feedback offered by AI-driven apps [28], our participants did not seem to be interested in this advantage. This could be due to a mistrust of the quality of rapid responses from AI, or a preference for a more reflective, considered feedback from human therapists. The complexities of using AI-driven apps are also highlighted by privacy issues and cultural competencies [55, 56].

Male participants and those who had already used a mental health app were more likely to prefer AI-driven apps to a human therapist, while those who had already experience with psychotherapy were more likely to prefer a human therapist. Age had no effect on the preference for AI-driven mental health apps. Our findings on the relationship between age and the use of innovative mental health treatments are not consistent with those of previous studies. Although Lincke et al. [24] reported that younger age significantly increased the probability of preferring online therapy rather than traditional therapy, we found no statistically significant relationships between age and preference for AI-based therapy. A CBCA of innovative skin cancer screening applications revealed that older individuals attach significantly greater importance to the traditional physician treatment than younger individuals [57]. However, the association found in our study between previous use of mental health apps and a greater preference for AI-based therapy is consistent with previous research. Prior experience with technology predicts the intention to use, as it leads to a richer understanding and concrete knowledge of the technology [40]. Our study revealed that men are more likely to use AI for mental health treatment than women are. While it was previously observed that more women than men use DiGA [53], Novozhilova et al. [58] discovered in their survey (*n* = 1506) that men tend to perceive AI in the healthcare sector as more capable than women. Other studies have demonstrated that there are no significant gender differences in willingness to utilize innovative mental health treatments [23, 25]. One possible reason for these disparities could be differences in sample size and recruitment. It is worth noting that some of the studies that did not find significant differences used smaller samples, such as that of Musiat et al. [25], who had a sample size of 460. Small sample sizes or non-representative samples might fail to capture subtle gender differences, leading to inconsistent findings across studies.

Our findings should be interpreted with several considerations in mind. One limitation is the potential lack of generalizability to other contexts. The relative importance of attributes was calculated in relation to all other attributes within this specific study, meaning that these importances may vary if other attributes were included or emphasized. This limitation is also present in studies that use more conventional methods for reporting attribute importance. To enhance the generalizability of our findings, future research should replicate this study in different countries and contexts. Additionally, applying this research design to other medical fields could help determine whether similar patterns emerge across different types of health treatments. Another limitation is the limited control we had over the experimental conditions, particularly whether participants fully engaged with the different attributes presented. Using eye-tracking technology to capture participants’ attention and gaze patterns could be a valuable method for addressing this issue in future research [59]. A further limitation of the study is that we did not set any specific time criteria for the completion of the survey in advance in order to exclude participants. However, we then set the time criteria for completing the CBCA based on a research paper by Sawtooth [48]. Although our sample comprised numerous participants with prior experience in psychotherapy, we lack insight into how current patients assess these treatment options. Another group of interest would be participants who are currently waiting for a place in psychotherapy. Notably, 41% of participants in our sample had experience with a traditional psychotherapy prior to the survey, a percentage that closely mirrors that of the general population [60]. By conducting a large-scale nationally representative study, we provided a comprehensive and insightful analysis of gender differences in preferences for mental health treatments, including AI-driven mental health apps.

## Conclusions

Our research reveals that the majority of participants preferred traditional human psychotherapy over AI-based therapy. They consistently prioritized affordable mental health treatment options operated by humans when presented with realistic alternatives, often basing their decisions on more superficial criteria rather than the content of the treatment. It is encouraging to note that individuals who have previously used a mental health app are more likely to express a preference for AI-based therapy. This suggests that they view AI-driven mental health apps as beneficial and efficacious. Nonetheless, those who have previously undergone traditional psychotherapy are less inclined to choose AI-based therapies. This finding highlights the need to consider how to engage individuals with existing mental health issues in using AI-driven mental health apps as a supplement or alternative to traditional psychotherapy. If AI-driven mental health apps are truly intended to compensate for the shortage of psychologists, they must be tailored to meet the most relevant criteria for the population, ensuring that they align with users’ preferences and needs. Despite potential advantages, significant doubts remain about whether AI-driven mental health apps can sufficiently address the growing demand for psychotherapy services and achieve widespread acceptance.

## Data Availability

The datasets generated and analyzed during this study are available in the zenodo repository [https://zenodo.org/records/10528219].
